# Effects of Crude Shea Butters and Their Polar Extracts on Singlet Oxygen Quenching and Against Rose Bengal-Induced HaCaT Cell Phototoxicity

**DOI:** 10.3390/molecules30061360

**Published:** 2025-03-18

**Authors:** Bertrand W. F. Goumbri, Olivia Jansen, Roland Marini Djang’eing’a, Michel Frederich, Rasmané Semdé, Touridomon Issa Somé, Sabine Danthine, Ange Mouithys-Mickalad

**Affiliations:** 1 Laboratoire de Recherche-Développement de Phytomédicaments et Médicaments, Institut de Recherche en Science de la Santé (IRSS/CNRST), Ouagadougou 7047, Burkina Faso; bertrand.bribera@gmail.com; 2 Laboratory of Pharmacognosy, Department of Pharmacy, Center of Interdisciplinary Research on Medicines (CIRM), University of Liege, 4000 Liege, Belgiumm.frederich@uliege.be (M.F.); 3 Laboratory of Pharmaceutical Analytical Chemistry (LPAC), Department of Pharmacy, Center of Interdisciplinary Research on Medicines (CIRM), University of Liege, 4000 Liege, Belgium; rmarini@uliege.be; 4 Centre d’Excellence Africain de Formation, de Recherche et d’Expertises en Sciences du Médicament (CEA-CFOREM), Université Joseph Ki-Zerbo, Ouagadougou 7021, Burkina Faso; rsemde@yahoo.fr; 5 Laboratoire de Toxicologie, Environnement et Santé, Université Joseph Ki-Zerbo, Ouagadougou 7021, Burkina Faso; ti_some@yahoo.fr; 6 Faculty of Gembloux Agro-Bio Tech, Food Science and Formulation, University of Liege, 5030 Gembloux, Belgium; sabine.danthine@uliege.be; 7 Centre for Oxygen Research and Development (CO2RD), Center of Interdisciplinary Research on Medicines (CIRM), University of Liege, 4000 Liege, Belgium

**Keywords:** shea butter, phenol, antioxidant, singlet oxygen, photoprotection, HaCaT

## Abstract

Shea butter (SB) is a raw material fat obtained from *Vitellaria paradoxa* C.F. Gaertn kernels. We investigated the direct and indirect protective effects of 10 traditional and industrial SBs and their polar extracts on cell-free systems using ABTS and DPPH radical scavenging assays as well as on singlet oxygen (^1^O_2_) produced by Rose Bengal (RB) photosensitization. Their effects against RB-induced HaCaT cell phototoxicity were also explored. A spectrophotometric assay and HPLC were performed to quantify and identify phenolic content, which was between 14.16 and 82.99 ppm pyrogallol equivalent. These variations could be due to the SB origin and extraction process. These polar fractions exhibited moderate DPPH and strong ABTS radical-scavenging activity. By applying the UV–visible technique, we demonstrated that SBs and their phenolic compounds behave as ^1^O_2_ quenchers in a dose-dependent manner. Moreover, using a UVR-like model after the irradiation of RB, both polar extracts and crude SB exhibited photoprotective effects, highlighting the indirect protective action. In acellular and cellular models, SB and its polar extracts can act as a free radical scavenger against reactive oxygen species and ^1^O_2_ quenchers. Due to the maximum absorbance of SB at 280 nm and the antioxidant effect of ^1^O_2_ quenching, SB polar extracts exhibit photoprotective properties.

## 1. Introduction

Solar ultraviolet radiation (UVR) induces skin damage, photoaging, and photosensitivity disorders [1]. There are three categories of UVR according to wavelength: long-wave Ultraviolet—UVA (315–400 nm), middle-wave UVB (280–315 nm), and short-wave UVC (100–280 nm). UVA and UVB radiations have important biological consequences for the skin [2,3]. UVB has high energy, and the stratospheric ozone absorbs approximately 90% of radiation. During long-term exposure, the remaining 10% of radiation can cause Deoxyribonucleic Acid (DNA) damage by producing a genotoxic effect via the production of reactive oxygen species (ROS) [4].

Titanium dioxide (TiO_2_) or titanium (IV) oxide, is a poorly soluble particle used as a white pigment. It is the natural oxide of Ti [5]. Likewise, TiO_2_ is an inorganic nanoparticle commonly used as a UV filter in sunscreen, lotions, and facial cream formulations [6]. According to Fujishima et al. [7], TiO_2_ has two main functions: absorbing and deflecting UVR, and semiconductor photocatalysis. To absorb and deflect UVR, an electron in the TiO_2_ valence band absorbs the photon energy and generates hydroxyl radicals (HO^•^). Then, the formation of ROS, such as superoxide (O_2_^•−^) and ^1^O_2,_ has been reported [8,9]. Against the harmful effects of UVR, sunscreens have been used as one of the main preventive measures. In sunscreens, UV filters protect the skin from the hazardous effects of UVR by absorbing or reflecting it.

On the human keratinocyte cell line (HaCaT) treated by TiO_2_, a clear dose-dependent increase in superoxide production, caspase 8 and 9 activities, and apoptosis was observed [10]. TiO_2_ nanoparticles’ photo-reactivity largely depends on their particle size, shape, and crystal structure, as anatase and rutile ones [11]. Smaller nano-TiO_2_ particles have higher phototoxicity, while anatase and amorphous forms show higher cytotoxicity than their rutile form, generating more ROS after UV irradiation [12]. HaCaT cells are widely used to study skin biology and differentiation [13]. This study used HaCaT cells to assess the photoprotective potential of several crude shea butters (SBs) and their polar fractions.

Shea (*Vitellaria paradoxa* C.F. Gaertn) is a tree from the *Sapotaceӕ* family that grows naturally across the Sahelo-Sahelian region in Africa, primarily in West Africa. Its subspecies, *V. paradoxa nilotica*, is found in East Africa. Shea kernels are used to extract shea butter, a versatile fat traditionally used for centuries in food, body care, pharmacopeia, and as a light source [14]. Shea butter extraction in Africa remains mostly traditional, with some semi-industrial and industrial methods [15].

The antioxidants naturally occurring in unsaturated oils are reducing agents that can block the peroxidation reaction, preventing the formation of hydroperoxides and peroxides [16]. They can also be photoprotectors in sunscreen formulations due to their anti-radical mechanisms, which apply to photo-oxidation. According to Fernandez et al. [16], vegetable oils are naturally rich in antioxidants, which protect them from oxidation. The degree of unsaturation of an oil defines its antioxidant capacity. The more unsaturated the oil, the more antioxidants it contains. Phenolic compounds, as well as tocopherol, ketonic, or aldehydic functional groups, provide antioxidant preservatives, either by protecting unsaturated fatty acids from oxidation during the initiation stage or by blocking the propagation stage by reducing the free radicals formed [16].

Shea butter is namely used in the cosmetic industry, firstly due to its particular higher unsaponifiable matter (triterpene alcohols, kariten, sterol, tocopherol, phenols, etc.), then to its melting point, and finally to its potential in the absorption of UVA and UVB radiation [17,18]. Skin protection properties against UVA–B radiation have been assigned mainly to cinnamic esters, which absorb at 250–300 nm both tocopherols and phytosterols [19,20]. According to Maranz et al. [21], the total polyphenol content (TPC) of the SB extracted with hexane was 35–915 ppm caffeic acid equivalent of the extract. The authors reported a variation in phenolic content according to the geographic origin of the SB. Kariten is an unsaponifiable content whose basic unit isoprene [(C_5_H_8_)n], mp 63°C, is a poly-isoprene hydrocarbon with rubber-like properties that could absorb UV. This characteristic confers some additional properties to SB to filter UV [22]. According to the authors, kariten content (2–5%) is related to the extraction process involved. However, there is limited literature on SB’s ability against ultraviolet radiation (UVR).

According to Packer, L. et al. [23], antioxidants prevent ROS from oxidizing or inhibit/quenching the formed ROS. Singlet oxygen contributes to the TiO_2_ photocatalytic reaction [9]. As ^1^O_2_ induces cellular toxicity, our study investigates ^1^O_2_ quenching related to two experimental devices: in vitro cell-free, and HaCaT cell models. This study aimed to investigate ^1^O_2_ quenching and direct the photoprotective effects against UVR of crude SB and their polar extracts. We focused on SB polar fractions which were evaluated simultaneously with the raw butter.

## 2. Results and Discussion

To perform analyses, we investigated ten research materials (see Section 3.1 Materials): seven traditional crude SBs, two industrial kinds of butter, and a kariten-rich extract (named kariten in our study).

### 2.1. Total Phenolic Content of Shea Butter and Kariten Samples

**Figure 1:**
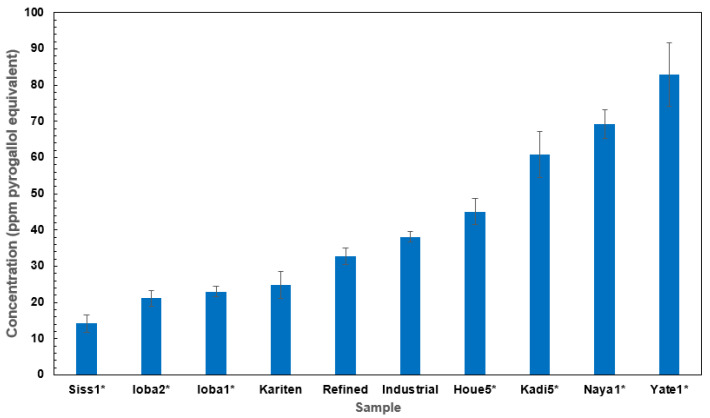
Total phenolic compound content of the different samples, expressed in ppm pyrogallol equivalent. Results were expressed as mean ± SD (see Table S1). Samples with * are traditional SB.

Regarding total phenolic content (TPC), the highest and lowest values were found among the traditional butter samples which are Yate_1_ (82.99 PGE) and Siss_1_ (14.16 PGE), as shown in Figure 1, and Table S1. The samples with the highest levels of TPC were Yate_1_ and Naya_1_, followed by Kadi_5_. The phenolic content varied considerably among traditional and industrial samples. These results showed that refined SB still contains phenolic compounds. However, according to Ainsworth and Gillespie [21,24], the FC method is non-specific to phenolic content because it reacts easily with oxidizable compounds, which are not necessarily phenolic compounds [25]. Several factors can impact TPC, such as material quality, the climatic conditions of cultivation, production processing, packaging, or storage [26]. For example, sample Yate_1_ had a particular extraction process involving no churning step, as described by Goumbri et al. [27]. Studies in ten African countries showed that the TPC of SB extracted with hexane ranged from 62 to 135 ppm, compared to 2100–9500 ppm for shea kernels, in gallic acid equivalent (GAE) [21]. According to the authors, phenolic content is related to the origin of the shea sample and the butter extraction process involved.

### 2.2. Antioxidant Activities of Crude Shea Butters and Their Polar Fractions

#### 2.2.1. Choice of Solvent to Solubilize Crude Shea Butter

Although cyclohexane is better at solubilizing SB, we chose to use ethanol (EtOH) for SB solubility in accordance with the test. Indeed, SB solubility in EtOH (Figure S1a) is similar to that in cyclohexane, Figure S1b, and the λmax was around 280 nm for both solvents. The compounds of interest (in the photoprotection test) are well soluble in EtOH, justifying its choice for further analyses. Another argument is its use as a solvent in cosmetic formulations due to its skin tolerance at concentrations of less than 25% [28].

#### 2.2.2. Antioxidant Activities

The DPPH radical and ABTS radical cation assays performed with crude SB at different concentrations showed negative results: no visible discoloration was observed. As a result, no antioxidant activity was directly found in crude butter for the ABTS and DPPH tests. The free radical DPPH° and radical cation ABTS°^+^ were not transformed into their stable forms, DPPH and ABTS, respectively. This could be due to raw butter’s shallow polyphenol content and potential interference with various butter compounds.

In contrast to raw butter, the tested polar fractions exhibited more potency on ABTS radical cation scavenging (Table 1) than on DPPH. The inhibition percentages for the highest final concentrations did not exceed 40% for DPPH, while for ABTS, values above 80% were reached. Most of the tested samples presented a dose-dependent inhibition effect which appears to link to TPC and phenol. Indeed, for ABTS scavenging, Naya_1_ and crude industrial butter (at the highest concentrations related to TPC) had the strongest inhibition levels (above 80%). The sample with the higher TPC (Yate_1_) had an ABTS inhibition of about 69%.

The relationship between TPC and antioxidant capacities (ABTS and DPPH average values) of the 10 samples was established using the Pearson correlation test at α = 0.05. A value of at least 0.50 indicated a statistically significant relationship, and values below 0.50 indicated no significant correlation.

In our study, the DPPH and ABTS values showed a moderate positive correlation with the TPC (R = 0.508 and 0.642, respectively) (see Table 2). Our findings were not similar to the results obtained by Zhang et al. [29] (no correlation with the TPC); and by Dudonné et al. [30], which found significant correlation in their study. Our results could be explained by the fact that samples are not all homogeneous due to their origin and extraction process. These results suggest that the presence of phenolic compounds moderately contributes to SB free radical scavenging capacities. A significant correlation was found between the ABTS and DPPH assays (R = 0.924), similar to the reports of Dudonné et al. [30] (R = 0.946), and could be explained by the fact that the antioxidant potential of phenolic compounds is directly linked to the molecular structure of phenols, depending on the number of aromatic rings and hydroxyl groups [30].

### 2.3. Photoprotection Effect of Raw Shea Butters and Their Polar Extracts on Singlet Oxygen

#### 2.3.1. Effect of Crude Shea Butters and Their Polar Fractions on Singlet Oxygen by Photosensitization of Rose Bengal (Acellular Model)

**Figure 2:**
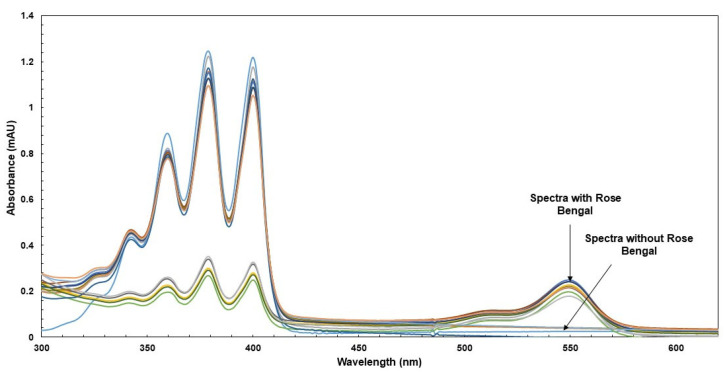
Influence of testing samples on the production of ^1^O_2_ induced by ADPA. The figure shows the spectra when the samples are in the presence or absence of RB, as illustrated by the Ioba_2_, Kariten, and TiO_2_ samples at T_0_ and T_300s_.

**Figure 3:**
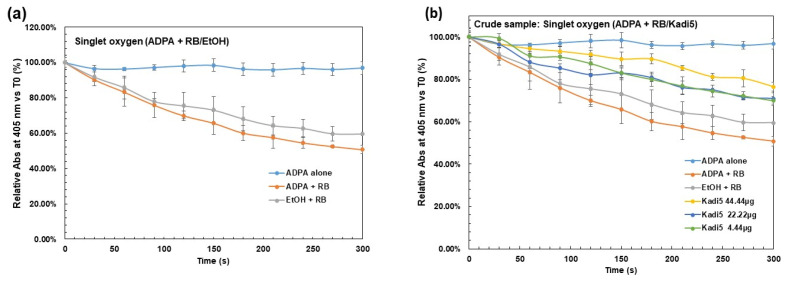

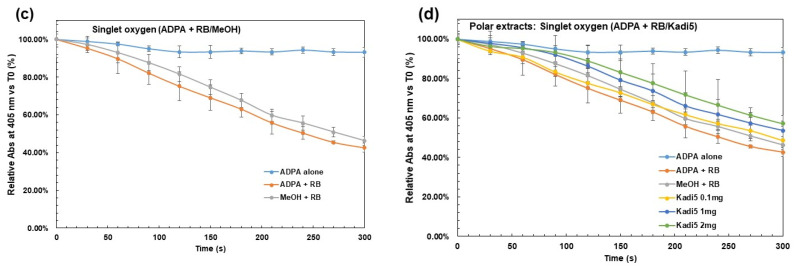
Effects of crude samples and their polar extracts by production of ^1^O_2_ upon RB photo-irradiation. Samples were studied at different concentrations versus EtOH and MeOH as solvent control at 0.44%. Absorbance is monitored at 400 nm, and the results are the mean ± SD (see Table 2) of two independent experiments. (**a**) Effect of ADPA alone and Ctrl (EtOH) on ^1^O_2_ production. (**b**) Crude sample effects on ADPA system. (**c**) Effect of ADPA alone and Ctrl (MeOH) on ^1^O_2_ production. (**d**) Polar extract effects on the ADPA system.

According to experimental conditions (see methodology), it appears that upon RB irradiation, the absorbance decreased over time at 400 nm, while in the absence of RB, no effect was observed either at 400 nm or at 550 nm (Figure 2). In contrast, when the raw sample was used at different concentrations in EtOH, a slowdown of absorbance was seen in comparison with controls (see Figure 3a,b), indicating a ^1^O_2_ scavenging capacity. Also, investigations recorded in MeOH showed similar behavior for polar extracts (Figure 3c,d).

From Figure 3, one can see that Ctrl (ADPA + RB) decreased more quickly than Ctrl (EtOH) and Ctrl (MeOH) for crude samples and polar extracts. Crude samples were tested at concentrations ranging from 4.44 to 44.44 µg/mL, and TiO_2_, used as a positive control, ranged from 0.44 to 22.22 µg/mL. For polar extracts, concentrations ranged from 0.003 to 0.369 ppm, corresponding to 0.1 at 2 mg of crude butter, as shown in Table S1. The photochemical reaction of ^1^O_2_, in the presence of RB, with tested samples was monitored over time according to the decay of ADPA absorbance at 400 nm. Figure 3b,d showed illustrative results. Results for all tested samples were recorded in Table 3, showing ADPA decay at the end of the reaction (300 s). Overall, tested samples exhibited dose-dependent ^1^O_2_ quenching effects. For crude SB, at the high concentration, Yate_1_ exhibited the maximum scavenging of ^1^O_2_, followed by Naya_1_ and Ioba_1_: 29.57 ± 0.92%–28.55 ± 0.62%–27.38 ± 1.04%, respectively more than TiO_2_ (14.23 ± 3.28%).

Results showed that traditional crude SBs were more ^1^O_2_ quenchers than industrial ones, which exhibited ^1^O_2_ inhibition ranging from 26.38 ± 0.92% to 19.19 ± 0.11%, respectively, for refined and kariten shea samples. For polar extracts, at the highest concentration, Ioba_2_ exhibited the maximum scavenging of ^1^O_2_ at 15.00 ± 2.61%, followed by Kadi_5_ and Yate_1_, more than crude industrial (12.53 ± 0.86%) and kariten (11.55 ± 0.65%).

These results could be explained by the fact that SB, as raw material, also contains phenols and a whole fraction of unsaponifiable matter, which exhibit biological properties [31], such as triterpene alcohols (65 to 75% of unsaponifiable content), the tocopherol (α: 112 µg/g of butter; y: 13 µg/g of butter; ß and δ), and the sterol [32,33].

Traditional SBs from Burkina Faso and their polar extracts exhibited more effects on ^1^O_2_ than industrial ones. According to our results, only raw samples exhibited an activity with a dose-dependent manner. These compounds are potent ^1^O_2_ quenchers, which could protect against UVA–B from UVR. However, the effects were not linked only to SB polyphenols. Antioxidants act by hydrogen donation to another molecule, yielding phenoxy radicals (on the benzene ring), resulting in an intramolecular hydrogen bond with the free hydrogen.

Regarding the effect of polar extract on the one hand and crude sample on the other, by the production of ^1^O_2_ upon RB photo-irradiation, we performed a single sample *t*-test to compare the means of relative absorbance of tested samples (see Table 3) on Ctrl ADPA. There was a significant difference between the means of tested polar extract and the crude sample on Ctrl ADPA (t (df) = 30, *p*-value = 1.09 × 10^−16^), (t (df) = 30, *p*-value = 1.69 × 10^−19^), respectively. Then, a two-sample *t*-test was also performed to compare the effect of tested samples by production of ^1^O_2_ upon RB photo-irradiation in polar extracts and raw samples. Results showed a significant difference in the effect of tested samples by production of ^1^O_2_ upon RB photo-irradiation between polar extracts (mean = 52.4, SD = 2.92) and crude samples (mean = 71.4, SD = 4.12); t (df) = 52.201, *p*-value < 2.2 × 10^−16^.

#### 2.3.2. Photoprotective Effects of Crude Shea Butter and Their Polar Extracts on Cells’ Viability by Photosensitization of Rose Bengal (Cellular Model)

- Inherent toxicity of crude shea butter on HaCaT cells

The results showed that EtOH, used as solvent control at a final concentration of 2%, displays no toxicity after 24 h (Figure S2a) and 48 h (Figure S2b) of experiments. Similar results were found with overall tested samples dissolved in EtOH at final concentrations ranging from 0.1 to 100 µg/mL, shown in Figure S2. The cell viability was over 60% after 24 h of incubation and over 90% after 48 h of incubation. The results showed that crude SB at the tested concentrations displayed no HaCaT cell toxicity after 24 h and 48 h of incubation. Based on these results, crude SB can be used for photoprotection investigations on HaCaT cells in EtOH.

- Protective effects of raw shea samples and their polar extracts against ^1^O_2_ production

**Figure 4:**
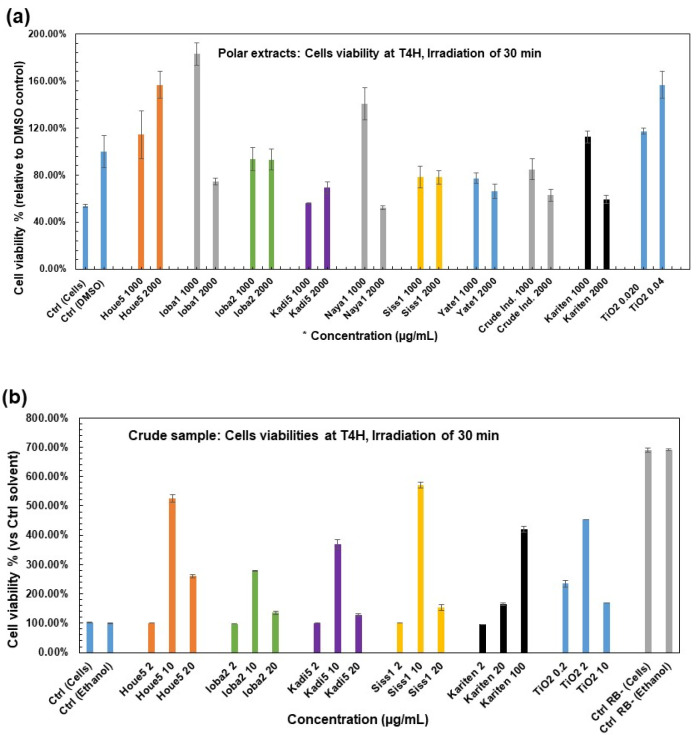
The application of the cellular model to investigate the effect of crude samples and their polar extracts vs. TiO_2_ on HaCaT cell viability. (**a**) Polar extracts and TiO_2_ were dissolved in DMSO. (**b**) Crude samples and TiO_2_ were dissolved in EtOH. The results are the mean of two independent experiments. The cell’s viabilities were recorded at 492 nm relative to solvent Ctrl (2% and 2%, respectively, for DMSO and EtOH). *: Concentration in raw butter equivalent and the correlation in TPC is shown in Table S1.

According to the different concentrations performed (Table S1), the results of the protective effects of the raw shea sample (Figure 4b) and its polar extracts (Figure 4a) showed that without RB, the cells were unaffected and continued to grow normally, showing even an increase very significantly to almost 700% viability, as illustrated in Figure 4b. This can be explained by the non-formation of ^1^O_2_ and, consequently, the absence of toxicity.

The cell viability was evidenced in crude butter after 4 h of incubation and growth from 96.23% to 570.75%, respectively, for kariten at 2 µg/mL and Siss_1_ at 10 µg/mL (Figure 4b). Comparatively, TiO_2_ viabilities ranged from 169.18% at 10 µg/mL to 453.13% at 2 µg/mL. The maximum viabilities were reported at 10 µg/mL concentrations for SB tested samples. However, kariten seems to provide dose-dependent protection. The results showed that TiO_2_ exhibited more protection than butter. These findings could be explained by the fact that TiO_2_ is a broad-spectrum UV filter, and in its microfine forms (20–50 µm), it offers photoprotection against UVB and UVA rays at 315–340 nm only, not covering the 340–400 nm range [34]. Also, Wright et al. [10] reported that the TiO_2_ particles (fine and ultrafine) had a positive effect on cell viability after 24 h of exposure upon UVC exposure at 254 nm for 30 min, 1 h, and 5 h, in contrast to the TiO_2_ nanoparticle. After 48 h of exposure, all forms of TiO_2_ harmed cell growth, but this was more pronounced with TiO_2_ nanoparticles.

Cell viability after 4 h of incubation ranged from 52.24% to 182.96% for polar extracts, respectively, for Naya_1_ (equivalent to 2 mg of butter) and Ioba_1_ (equivalent to 1 mg of butter). The results for TiO_2_ were from 117.34% to 156.80% (Figure 4a). Polar compounds provide better protection than TiO_2_ at the highest values for this experiment in an aqueous medium, even though irradiation of TiO_2_ in an aqueous suspension and MeOH generated ^1^O_2_ and hydrogen peroxide [35]. Some studies have shown that TiO_2_ (at 1.0 g/L) has a low potential toxic effect only after long-term exposure. The cytotoxic effect only appears at very high concentrations, reducing cell viability after seven days of exposure [36]. These results agree with our findings. Cell viability levels were diversely distributed and not necessarily dose-dependent, except for Houe_5_. However, the results showed that the lowest concentrations provided the best protection for Ioba_1_, Naya_1_, crude industrial butter, and kariten.

Based on photoprotection results for raw butter, a dose-dependent effect, a one-sample *t*-test was performed to compare Ctrl Ethanol against the tested crude sample mean (see Figure 4b). There was a significant difference between the means of the tested crude sample on the Ctrl Solvent (t (df) = 15, *p*-value = 0.00748).

### 2.4. LC Analyses of Shea Butter Polyphenols Content

- Analyses of polar compounds in polar fractions and quantification by RP-HPLC

Four polar constituents were identified, namely, gallic acid, protocatechuic acid, cinnamic acid, and quercetin (see Figure S3a), using their retention time (Rt) compared to those of standards, and their UV spectrum using an internal natural products HPLC-UV/DAD database. The identification was then confirmed by comparison of the Rt and UV spectrum for each pre-identified peak with pure compound individually injected into the HPLC system.

Our results showed that a few compounds were widely distributed across the samples. Gallic acid was only found (in low amounts) in kariten and Yate_1,_ with contents below the GAE limit of quantification (LOQ). Cinnamic acid was the most representative constituent as it is present in almost all the samples and ranged from 0.00 to 46.26 ppm GAE. For kariten, a non-identified compound was eluted at around 40 min, with an Rt similar to the cinnamic acid, but with a different UV spectrum. Phenol contents ranged from 0.972 to 46.888 ppm GAE, as shown in Table 4. However, some compounds were below the LOQ in GAE (Table 4). First, compared with the colorimetric assay, the lowest levels reported by HPLC could be linked to the different steps in the traditional butter extraction processes. Next, it is also linked to the fact that we do not quantify all the peaks in LC analysis. Fewer compounds are considered than in TPC with the FC method, where a totum is quantified. Flavonoids, for example, were not quantified by LC but reacted in the TPC using the FC method. Likewise, according to Maranz et al. [21], the extraction of SB with hexane is responsible for a 90–98% depletion of phenol. Phenol content depends on the extraction technique [31], and others suggested modifying extraction and refining processes to obtain higher levels. High temperatures in the extraction process can destroy antioxidants [37]. For traditional SB, produced by no standardized processes, more parameters, such as moisture content, insoluble impurities, free fatty acids, and peroxides, can decrease their shelf-life, thus their polyphenol compounds, in contrast to industrial ones.

The HPLC analysis also revealed other peaks that could not be identified from the available spectral database and Rt values. Most of these compounds present a UV-spectrum with maxima of absorbance between 200 and 300 nm. Interestingly, all chromatograms show three non-identified peaks (named *C8*, *C9*, and *C10*) at Rt = 68–69 min. DAD analysis of their UV spectra showed that they all three overlayed with the cinnamic acid UV spectrum, as shown in Figure S3b. These three peaks were found in all the samples and eluted between 68 min and 70 min, corresponding to 100% acetonitrile in the gradient mobile phase. According to their higher Rt in RP-HPLC (Rt cinnamic acid = 40.3 min), it can reasonably be deduced that they could correspond to 3 cinnamate derivatives, bearing a cinnamate chromophore combined with a less polar moiety. This could match with some of the literature data describing the presence of triterpenes alcohol cinnamate esters in SB.

A study carried out on Nigerian SB (hexane-extracted) isolated and identified four triterpene cinnamate compounds: α-Amyrin cinnamate, β-Amyrin cinnamate, lupeol-cinnamate, and butyrospermol cinnamate. In addition to cinnamates, four other triterpene acetates have been isolated and identified [38]. Later, a similar study focusing on shea butter (hexane-extracted) from seven African countries re-identified the same cinnamate triterpene compounds and found that triterpene cinnamate compounds predominated over acetate triterpenes [39]. A review on the use of SB and its derivatives in cosmetics also reported the presence of these triterpene cinnamate esters at significant levels with reported main values of: α-Amyrin cinnamate (29.3%), β-Amyrin cinnamate (7.6%), lupeol-cinnamate (9.0%), and butyrospermol cinnamate (14.8%), relative to the total triterpene esters [40]. Although the presence of these compounds could not be strictly confirmed in our HPLC-UV/DAD analysis, we can state that, according to their UV spectra, and as cinnamate derivatives, compounds *C8, C9* and *C10* probably contribute to the photoprotective potential of the SB samples analyzed in this study. Indeed, according to Alesandra R. et al. [41], flavonoids and cinnamate derivatives are considered as natural photoprotectors, with a preference for cinnamate derivatives used as UVB–UVA filters in the (310–325 nm) wavelength. The photoprotective effects of some cinnamate derivatives are well documented and some are used in commercial sunscreen products.

Regarding the polar constituents detected in the polar fractions, only Naya_1_ presented compounds at 47 min and 55 min. Also, samples Naya_1_ and Yate_1_ had the most significant levels in TPC and were among the samples with highest inhibition percentages in the ABTS/DPPH assays. This could also be related to the detection of quercetin in these samples, but at non-quantifiable concentrations (<LOQ). According to Gilaberte and González [42], quercetin is the flavonoid with the most potent antioxidant properties. When incorporated into some topical formulations, it inhibits damage caused by UV-B radiation in animals. Secondary plant metabolites such as flavonoids and cinnamic acids are considered natural photoprotectors [41]. Found in SB, flavonoids are a group of polyphenols that absorb significantly in the UVA–UVB range due to their molecular structure and double bonds [43]. However, according to some authors [44,45], flavonoids act by absorbing UV rays, by direct and indirect antioxidant action by trapping the ROS generated after UV irradiation, by an anti-inflammatory effect by contributing to the reduction of inflammation caused by irradiation, by immunomodulatory actions, and by DNA protection and repair.

Kariten significantly reduced the ABTS radical by 81.29% at its highest concentration (1.49 ppm). The polar fraction’s free radical scavenging activities by reducing ABTS and DPPH confirmed that SB phenolic compounds exhibited antioxidant activity. At experimental concentrations, crude SB and its polar fraction protected HaCaT cells from ^1^O_2_ generated after irradiation at 550 nm in the cellular model. In the acellular model using the ADPA + RB system, findings also provided evidence of the inhibition of ^1^O_2_ produced by irradiation in experimental conditions. Shea butter is used as an active ingredient in sunscreen formulations. The properties of sunscreens have been described in several studies [42,46], meaning that SB may act through a combination of physical and chemical actions. Due to its lipophilic properties, SB can absorb, reflect, and/or disperse emitted radiation. Its high unsaponifiable matter content [15], could provide SB with some direct and indirect antioxidant activities against phototoxicity by trapping ^1^O_2_ and inducing intrinsic cytoprotective reactions, respectively [42].

## 3. Materials and Methods

### 3.1. Materials

Two types of samples as shown in Table 5 were analyzed: seven crude traditional SB samples (from different origins in Burkina Faso, a West African country), two industrial butters (one crude and one refined, from IOI Loders Croklaan, Maasvlaktle, The Netherlands), and a kariten-rich extract, from Belgium. The traditional crude butter samples were selected according to their extraction process and physicochemical quality, as described previously by Goumbri et al. [27].

#### 3.1.1. Materials for Total Phenol Content

TPC was performed with the polar extract obtained from each crude sample: seven traditional SBs, two industrial SBs, and one kariten-rich one.

#### 3.1.2. Materials for Antioxidant Test

Regarding the antioxidant test, two kinds of samples were used: crude samples, and their polar fractions.

#### 3.1.3. Materials for Photoprotection Test

As previously mentioned, crude samples and their polar fractions were used to perform the photoprotection test. Cellular investigations were made with Human keratinocytes (HaCaT cell line).

#### 3.1.4. Materials for Polyphenol Identification and Assay

Polyphenol identification and assay were performed with the polar extract obtained from each crude sample: seven traditional SBs, two industrial SBs, and one kariten-rich one.

#### 3.1.5. Cell Culture and Media

Keratinocytes (HaCaT cell line) were obtained from ATCC (Rockville, MD, USA) grown on 96-well plates (passage 39 to 45) in Dulbecco Modified Eagle’s medium (DMEM) supplemented with 10% fetal bovine serum (FBS), and antibiotics (penicillin 100 U/mL, and streptomycin 0.10 mg/mL. Cells were incubated at 37 °C in a humidified atmosphere containing 5% CO_2_, counted, and distributed at the concentration of 10,000 cells/well [47,48].

#### 3.1.6. Chemicals

Analytical-grade chemicals, standards, and reagents from commercial sources were used to perform the analysis. Titanium (IV) oxide, nanopowder, <100 nm particle size (BET), ≥97% were from Sigma Aldrich (St Louis, MA, USA). Hank’s Balanced Salt Solution (HBSS), Phosphate buffered saline (PBS), and Pyrogallol were all from Sigma Aldrich (Steinheim, Germany). A quantity of 3-(4,5-Dimethylthiazol-2-yl)-5-(3-carboxymethoxyphenyl)-2-(4-sulfophenyl)-2H-tetrazolium (MTS) was purchased from Promega Corporation (Madison, WI, USA). Dimethyl sulfoxide (DMSO), 2,2-Diphenyl-1-picrylhydrazyl (DPPH), and 9,10-Anthracenedipropanoic acid (ADPA) were obtained from Merck (Darmstadt, Germany). A quantity of 3-ethylbenzothiazoline 6-sulfonic acid (ABTS) was from Fluka (part of Sigma-Aldrich, Saint Louis, MA, USA), and Phosphomolybdotungstic reagent (Folin–Ciocalteu reagent), Methanol and Ethanol were from VWR Chemicals (Leuven, Belgium). The reference standards, such as Cinnamic acid, Catechin, and analogs, were purchased from Extrasynthese (Genay Cedex, France), and Gallic acid was purchased from Sigma Aldrich (Buchs, Switzerland).

### 3.2. Methods

- Sample preparation for photoprotective and antioxidant investigation (solution A)

First, a comparative analysis of EtOH (solution A1) and cyclohexane (solution A2) was carried out to choose the solvent that would provide the best solubility for the compounds of interest in crude SB. The importance of this step was to select a solvent that is non-cytotoxic while solubilizing crude SB. Solution A was prepared by dissolving 1.0 g of the melted sample in 100 mL of the appropriate solvent (ethanol or cyclohexane) in a volumetric flask, mixed for 5 min, and filtered through a 0.45 µm Millipore. A 1.0 mL aliquot was transferred to a 10 mL volumetric flask for absorbance measurement, and the volume was adjusted with the appropriate solvent. The absorbance of the sample in solution was recorded in three replicates at a wavelength between 280 and 400 nm (UVA–B range) using a spectrophotometer (Diode array, Agilent 8453, Agilent (Hewlett–Packard, Wald Bronn, Germany). Then, to investigate the photoprotective ability and antioxidant effects of raw samples, stock solution A1 or solution A2 was prepared, and dilutions were carried out.

- Polar fraction preparation (solution B)

To quantify TPC, polar fractions (solution B) were prepared from each sample according to some authors [21,24]. An amount of 500.0 mg of melted sample was weighed and dissolved in 5 mL of hexane. This hexane solution was successively extracted three times with 2 mL of a methanol–water (60:40 *v*/*v*) solution (liquid–liquid extraction). The three hydro-methanolic fractions were collected, combined, and evaporated to dryness at 40 °C using a Multivapor P-6 from Buchi. Dry residue was dissolved in 1.0 mL of methanol (solution B1) and filtered through a 0.45 µm Millipore. These methanolic solutions of the polar fractions obtained from each sample were used for the colorimetric assay, the HPLC analysis, and further antioxidant screening activities. To investigate polar fraction photoprotective effects, the same procedure was applied to each sample, but the dry residue was dissolved in 1.0 mL of DMSO (solution B2).

#### 3.2.1. Methods for Total Phenol Content

The solution B1 was used to perform TPC by colorimetric analysis using the FC reagent method, according to some authors [21,24]. In a volumetric flask of 5.0 mL, an aliquot of 200 µL of solution B1 was mixed with 125 µL of FC reagent and 2.5 mL of Milli-Q water. To avoid the air oxidation of phenol [24], 1 mL of saturated sodium carbonate decahydrate was added after 3 min, then mixed. The content was made up of volume with alkali. After 1 h of incubation, the absorbance was measured at 730 nm against a blank prepared in the same conditions without a tested sample, using a UV-spectrophotometer (Hitachi U-2910 UV–vis double beam, Hitachi Group Companies, Tokyo, Japan).

Spectrophotometric standard calibration was performed with pyrogallol to calibrate the concentration and prepared as follows: A stock solution of pyrogallol (0.25 mg/mL) was prepared in MilliQ water and diluted to obtain solutions of 0.502, 1.255, 2.510, and 5.020 µg/mL. TPC was calculated as PGE using the calibration curve. Analysis was performed in triplicate.

#### 3.2.2. Methods for Antioxidant Test

Both solutions A (for raw butter) and B1 (for polar fraction) were used at different concentrations in their appropriate solvent to perform antioxidant activity. Antioxidant activity screening of crude SBs and their polar extracts was performed by free radical DPPH^o^ and radical cation ABTS°+ scavenging decolorization assays, according to the optimized method [49,50]. For this, two controls were performed: a positive control with no added solvent (100 µL DPPH/ABTS) and a solvent control with 2 µL MeOH + 98 µL DPPH/ABTS. Three replicates were made using a 96-well microplate reader (Thermo Labsystem, Vantaa, Finland).

- DPPH radical-scavenging activity

The free radical DPPH° (purple color in solution) is reduced to DPPH-H (light yellow color) by a proton-donating compound with anti-radical properties. A 0.26 mg/mL DPPH test solution in absolute MeOH was prepared and mixed for 30 min in the amber flask. Dilution was made to obtain absorbance around 0.75 at 510 nm using a spectrophotometer (Agilent 8453, Agilent Technologies Deutschland GmbH). For the assay, a defined concentration was added to the DPPH working solution (final volume at 100 µL) in the 96-well plate and immediately read for the first time. For the polar fraction, the concentration corresponded to 2, 1, 0.5, and 0.05 mg of butter, respectively. Regarding crude butter, 200, 100, and 10 µg/mL were used as tested solutions. Then, the plate was incubated for 60 min in dark conditions prior to another reading. During the reaction, and in the presence of a DPPH° free radical, a proton from the antioxidant compound in the sample is transferred to the free radical, transforming it into a stable DPPH. The absorbance decreases until the hydrogen-donating antioxidant is exhausted. To get more data for statistical analysis, absorbance measuring was carried out at 510 nm using a Multiskan Ascent spectrophotometer (Thermo Labsystem, Vantaa, Finland). The percentage of DPPH° inhibition (I%) is calculated according to the Equation (1):

(1) $$I\%=\frac{Ablank-Asample}{Ablank}\times 100$$

where *A_blank_* = absorbance of the solvent control; *A_sample_* = absorbance of the tested sample.

- ABTS radical-scavenging activity

The ABTS assay was based on the method previously reported [49,50]. The oxidation of ABTS with sodium persulfate will generate a radical mono-cation of (ABTS°^+^), which will be reduced in the presence of such electron/hydrogen-donating antioxidants. A sodium persulfate (4.6 mg/mL) aqueous solution was mixed with an ABTS (26.5 mg/mL) aqueous solution by dissolving *v*/*v*. Then, the resulting mixed solution was stored overnight in the darkroom to obtain a dark-colored solution. The working solution was made up by diluting the stock with MeOH to obtain an absorbance between 0.70 and 0.80 at 734 nm using a spectrophotometer (Agilent 8453, Agilent Technologies Deutschland GmbH). In the presence of a potential antioxidant compound, radical cation ABTS^o+^ is transformed into its stable form, ABTS, by decolorizing the solution from blue/green to a neutral color. To measure the ABTS reducing the capacity of the sample, the absorbance was measured at 740 nm after 60 min, and the inhibition percentage was calculated in the same way as for the DPPH test using Equation (1). Regarding polar fraction, the analyses consisted of adding a defined concentration corresponding to 3, 2, 1, 0.5, and 0.05 mg of butter to the ABTS working solution (final volume at 100 µL). The same analytical conditions were applied to raw butter.

However, according to the guidelines to accurately estimate EC_50_ [51], this value could not be calculated because most of the samples did not have sufficient measurement points to be able to determine them properly.

#### 3.2.3. Methods for Photoprotection Test

- Designing experimental devices

**Figure 5:**
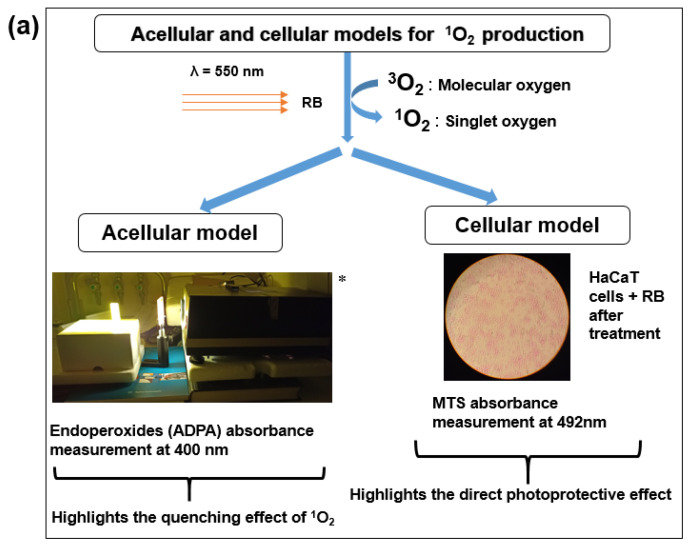

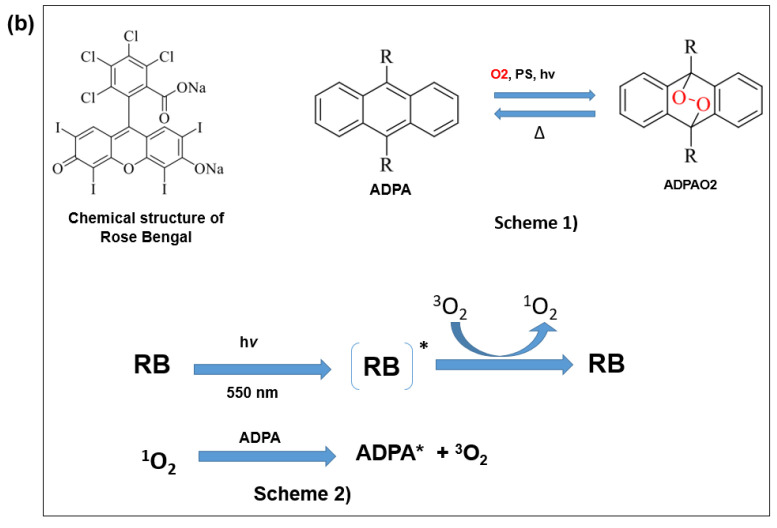
Effect of ^1^O_2_ photosensitized by RB on cell viability. (**a**) Cellular and acellular experimental devices optimized by irradiation at λ550 nm to produce ^1^O_2_. (**b**) ADPA model: ADPA reacts with ^1^O_2_ to form endoperoxide, after irradiation. (RB)* is an activated species produced after RB’s illumination. Schemes (1 and 2) show the ^1^O_2_ production with RB.

To establish comparative studies between crude SBs and their polar extracts against a UVR-like model using RB photosensitization, two experimental devices were applied (see Figure 5a and Figure S4) and optimized: an acellular model was used to investigate ^1^O_2_ quenching, and a cellular one to evidence the direct photoprotective effect of studied samples against photosensitized RB-induced phototoxicity of HaCaT cells (measurement of cell viability by MTS assay).

- In vitro experimental design to investigate ^1^O_2_ quenching capacity

The quenching effects were studied by adding RB to the ADPA solution before irradiation at 550 nm. Absorbance was recorded each 30 s, during 300 s at 400 nm in a spectrophotometer. In the ADPA system, when irradiated at 550 nm, the RB switches to an excited state, releasing ^1^O_2_, followed by consumption of ADPA at 400 nm.

An experimental working solution was prepared: 8 mg of ADPA was mixed in 12 mL of phosphate buffer at pH 7.0. The ^1^O_2_ was produced upon RB photosensitized by visible light from a slide projector (Pradovit RA 150, Leitz Wetzlar, Germany), equipped with a cut-off filter (Schot OG 500), which is used to select a specific irradiation wavelength at 550 nm [52]. The measurements were recorded in a UV-spectrophotometer under light conditions. A sample containing RB and ADPA was irradiated by a light source at λ550 nm at room temperature in a quartz cell and transferred immediately into the spectrophotometer for measuring the absorbance every 30 for 300 s. Buffer (phosphate buffer, pH 7.0) was used as a blank to calibrate before each measurement. ADPA reacts with ^1^O_2_ to produce an endoperoxide whose spectral characteristics show an absorbance decrease at 400 nm (see Figure 5b).

- Crude Shea Butter and Its Polar Fraction Assay on HaCaT Cell Viability

For the first time, the intrinsic effect of raw samples was investigated on HaCaT cells. Afterward, increasing concentrations of solution A (0.1, 1.0, 10, and 100 µg/mL) were tested on RB-induced HaCaT cell phototoxicity according to some authors [10,53]. TiO_2_ was used as a positive control for this assay to compare with tested samples. Nano powder of TiO_2_ was suspended in DMSO and dispersed using a sonicator for the 90 s at 5 s intervals to prepare TiO_2_ stock suspension at 1% m/v. Analysis was performed in a 96-well microplate reader in three replicates by two independent assays. Before each experiment, fresh solutions, A, B2, and TiO_2_ stock solutions, were prepared, and dilutions were made up with appropriate solutions. HaCaT cells were immediately treated for experiments according to the method described by authors [10,54].

***Crude shea butter’s inherent toxicity:*** *The* effects of crude SB on HaCaT cell viability were performed using MTS assay. Cytotoxicity was carried out on the one hand after treatment during 24 h (Figure S2a) and on the other hand after treatment during 48 h (Figure S2b). Cells were incubated with SB-tested samples for 24 h and 48 h to determine the toxicity of raw samples towards HaCaT. MTS assay was performed to measure cell viability at T_0_, T_2h_, and T_3h_. Briefly, the cells were washed with HBSS buffer before being treated with different concentrations of the tested sample. In each well containing 100 µL of the adherent cells in buffer, 2 µL of the tested sample was added.

HaCaT cell treatment and RB irradiation to investigate sample photoprotection ability: The effect of the tested samples (raw butter and polar extracts) against a UVR-like model at 550 nm radiation was investigated at different concentrations (Table S1). To avoid MeOH cytotoxicity on HaCaT cells, the stock solutions of the polar dry extracts were prepared by dissolving each extract in DMSO (see solution B2), and dilutions were made up. The aim was to induce cellular phototoxicity, which can be reversed in the presence of tested samples. TiO_2_ was used as a positive control at 0.02 to 0.04 µg/mL and 0.2 to 10 µg/mL for phenolic and crude SB assays. Both experiments were recorded according to cell and solvent controls at which RB was added (2% for DMSO and EtOH, respectively).

Protection against ^1^O_2_ induced phototoxicity: The formation of ^1^O_2_ under the UV irradiation of aqueous solutions, containing aqueous RB solution at a final concentration of 10^−5^ M, was assessed spectroscopically by MTS assay (Agilent 8453 UV–visible spectrophotometer). As for the inherent toxicity assay, 2 µL of each test solution (solution A at different concentrations) was added to 100 µL HaCaT cells (10,000 cells/well) in the presence or absence of 2 µL of RB photosensitizer and compared to the solvent control (DMSO). The 96-well plate was covered, incubated for 10 min at 37 °C, and then irradiated for 30 min according to the experimental procedure previously described (Figure 5a and Figure S4). After irradiation, the cells were washed twice with HBSS, and fresh buffer was added (100 µL/well). To measure cell viability, 10 µL of MTS was added to each well, and the absorbance was immediately measured at 492 nm with a Multiskan Ascent spectrophotometer. Viability measurements were carried out after each incubation time (1 h, 2 h, 3 h, and 4 h) at 37 °C. A normal control (HaCaT + RB) and solvent control were performed to establish cell phototoxicity. In the same plate, controls were performed in the same conditions but without RB taken as control (no RB).

#### 3.2.4. Methods for Polyphenols Identification and Assay

The identification and quantification of phenolic and organic acid compounds were performed using an internal HPLC method, optimized in the Laboratory of Pharmacognosy (Department of Pharmacy, University of Liege, Belgium). An aliquot of solution B1 was injected (10 µL) into the HPLC system (Agilent Technologies 1290 Infinity II, Santa Clara, CA, USA) equipped with a diode array detector (1290 DAD). Chromatograms were acquired at 210, 235, 254, 280, 330, and 350 nm, and the UV spectrum (from 190.0 to 400.1 nm) was also recorded for the detected compounds.

The system included a column Luna PFP packed with 5 µm, 250 mm length × 4.6 mm i.d., (Phenomenex), maintained at 30.0 °C. The gradient of mobile phase included acetonitrile (A) and aqueous formic acid 0.1% at pH 2.54 (B). Elution was performed at a flow rate of 1.0 mL/min, using the following (A) proportions (*v*/*v*): 15–18% at 0–15 min; 23–30% at 25–37 min; 40–50% at 42–52 min; 80–100% at 57–62 min; and 100% at 72 min. Shea phenol compounds were quantified at 280 nm using the standard gallic acid, in GAE. The compounds were identified using an internal HPLC/UV database (Laboratory of Pharmacognosy, University of Liège), combining Rt and UV spectrum data.

## 4. Statistical Analysis

The results are reported as mean ± SD of at least three replicates/treatments. Statistical procedures and graphical analysis were performed using Microsoft Excel 2019 software. Correlation (R) between in vitro parameters, TPC, ABTS, and DPPH values of different samples were made using Pearson’s method with the Software RStudio (version 2023.06.1). Then, acellular and cellular photoprotective data were assessed using the Student’s *t*-test (one-sample and two-sample *t*-test) to analyze the effects of Ctrl on tested samples, with a *p*-value of less than 0.05 considered significant.

## 5. Conclusions

According to acellular and cellular assays, the effects of different shea butter samples and their polar extracts have been investigated on ^1^O_2_ quenching following Rose Bengal sensitization. First, the antioxidant activity of polar extracts was evaluated for their ability to scavenge both DPPH radical and ABTS radical cations. We demonstrated that shea butter and its polar extracts exhibited antioxidant activities. However, crude material is more effective on ^1^O_2_ quenching than its polar fraction. Moreover, traditionally extracted butter displays a stronger effect on ^1^O_2_ quenching than industrial ones (crude and refined). According to the cellular model, using HaCaT cells, the highest concentrations of traditional butters tested did not necessarily confer the best cellular protection against ROS generated by photo-irradiation. Results highlighted shea butter’s direct and indirect photoprotective effects, including for its polar fractions against solar radiation. Therefore, the reported photoprotective effect of shea butter could be attributable to synergistic physical and chemical effects: hydration of the bi-lipidic double layer, and the effect of the unsaponifiable fraction. However, this study was limited due to the lack of molecular mechanisms investigation. For further analysis, it is therefore planned to extend this study by investigating compounds that have not been identified by RP-HPLC. Likewise, the ROS content could also be investigated to gain more mechanistical information.

## Figures and Tables

**Table 1:** Effects of shea polar fractions on ABTS and DPPH free radical scavenging. Tested concentrations are expressed in mg SB/mL calculated from the respective extraction yields of the polar fractions (see Table S1).

Sample	Concentration (mg/mL)	DPPH: I (%) at 60 min	ABTS: I (%) at 60 min
Crude Industrial	0.05	5.07 ± 0.17	7.85 ± 0.31
Crude Industrial	0.5	3.85 ± 0.12	13.60 ± 0.32
Crude Industrial	1	6.82 ± 0.21	21.15 ± 0.46
Crude Industrial	2	15.44 ± 0.28	27.29 ± 0.49
Crude Industrial	3	-	82.01 ± 0.15
Refined Industrial	0.05	4.40 ± 1.06	10.42 ± 0.96
Refined Industrial	0.5	7.17 ± 0.26	8.16 ± 0.50
Refined Industrial	1	5.24 ± 0.06	9.82 ± 0.46
Refined Industrial	2	10.84 ± 0.72	5.18 ± 0.44
Refined Industrial	3	-	56.12 ± 0.75
Kariten	0.05	8.39 ± 1.18	6.19 ± 1.22
Kariten	0.5	4.55 ± 0.56	7.85 ± 0.61
Kariten	1	7.34 ± 0.49	16.01 ± 0.40
Kariten	2	25.13 ± 0.71	39.02 ± 0.14
Kariten	3	-	81.29 ± 0.25
Houe_5_	0.05	2.62 ± 1.17	2.11 ± 0.98
Houe_5_	0.5	2.80 ± 0.21	3.63 ± 0.29
Houe_5_	1	6.64 ± 0.26	11.33 ± 0.35
Houe_5_	2	17.86 ± 0.92	27.59 ± 0.21
Houe_5_	3	-	73.81 ± 0.15
Ioba_1_	0.05	5.24 ± 1.54	2.11 ± 0.60
Ioba_1_	0.5	3.67 ± 0.40	4.68 ± 0.61
Ioba_1_	1	6.47 ± 0.74	7.10 ± 0.79
Ioba_1_	2	6.28 ± 0.85	12.35 ± 0.25
Ioba_1_	3	-	66.33 ± 0.26
Ioba_2_	0.05	0.70 ± 0.25	1.21 ± 0.36
Ioba_2_	0.5	3.67 ± 0.67	4.23 ± 0.90
Ioba_2_	1	11.19 ± 1.46	12.08 ± 1.35
Ioba_2_	2	15.17 ± 0.21	16.46 ± 0.34
Ioba_2_	3	-	55.54 ± 0.30
Kadi_5_	0.05	4.55 ± 0.10	11.78 ± 1.11
Kadi_5_	0.5	12.06 ± 0.47	19.64 ± 0.57
Kadi_5_	1	19.06 ± 0.38	35.05 ± 1.32
Kadi_5_	2	26.48 ± 0.49	46.8 ± 0.56
Kadi_5_	3	-	69.21 ± 0.21
Naya_1_	0.05	7.34 ± 0.58	21.00 ± 2.72
Naya_1_	0.5	13.99 ± 0.36	28.55 ± 1.01
Naya_1_	1	21.68 ± 0.25	44.71 ± 0.75
Naya_1_	2	35.64 ± 1.06	67.99 ± 0.31
Naya_1_	3	-	82.88 ± 0.06
Siss_1_	0.05	8.92 ± 1.81	5.14 ± 0.81
Siss_1_	0.5	4.90 ± 0.70	3.32 ± 0.55
Siss_1_	1	4.20 ± 0.76	4.68 ± 1.10
Siss_1_	2	5.48 ± 0.78	2.44 ± 0.29
Siss_1_	3	-	45.18 ± 0.56
Yate_1_	0.05	0.35 ± 0.20	2.72 ± 0.40
Yate_1_	0.5	3.32 ± 0.25	4.53 ± 0.49
Yate_1_	1	6.82 ± 0.06	13.90 ± 0.35
Yate_1_	2	17.06 ± 0.42	32.16 ± 0.32
Yate_1_	3		69.21 ± 0.12

The inhibition for DPPH and ABTS was established after 60 min, and results showed the mean ± SD (see Table S1) of triplicates with two independent assays.

**Table 2:** Correlation coefficient between in vitro parameters (ABTS, DPPH, ADPA model, and TPC).

	DPPH *	ABTS *	TPC
DPPH	1	0.924	0.508
ABTS	0.924	1	0.642
TPC	0.508	0.642	1

*: Average values are used to establish correlation.

**Table 3:** Effects of crude samples and their polar extracts on the production of ^1^O_2_ upon RB photo-irradiation. Absorbance is monitored at 400 nm showing ADPA decay at the end of the reaction (300 s) for all tested samples.

Sample	Polar Extracts		Crude Sample	
	Concentration (ppm)	Relative Abs at 300 s (%)	Concentration (µg/mL)	Relative Abs at 300 s (%)
	ADPA Alone Ctrl (ADPA + RB) Ctrl (MeOH)	93.22 ± 2.56	ADPA Alone	96.83 ± 2.56
		42.59 ± 2.31	Ctrl (ADPA + RB)	50.75 ± 2.31
		46.27 ± 2.27	Ctrl (EtOH)	59.48 ± 1.06
Houe_5_	0.010	50.82 ± 1.11	4.44	67.01 ± 2.49
Houe_5_	0.100	53.54 ± 2.07	22.22	70.98 ± 1.06
Houe_5_	0.201	55.39 ± 0.23	44.44	76.02 ± 2.25
Ioba_1_	0.005	50.13 ± 2.77	4.44	66.23 ± 1.01
Ioba_1_	0.051	49.00 ± 4.98	22.22	73.23 ± 2.1
Ioba_1_	0.102	50.34 ± 1.91	44.44	78.13 ± 1.04 °
Ioba_2_	0.005	48.43 ± 1.05	4.44	67.07 ± 1.05
Ioba_2_	0.047	51.79 ± 1.86	22.22	71.03 ± 2.80
Ioba_2_	0.094	57.59 ± 2.61 ***	44.44	74.00 ± 3.01
Kadi_5_	0.013	48.60 ± 1.90	4.44	69.84 ± 2.1
Kadi_5_	0.135	53.57 ± 7.70	22.22	70.90 ± 2.64
Kadi_5_	0.270	57.15 ± 1.35 **	44.44	76.46 ± 2.02
Naya_1_	0.015	49.19 ± 6.60	4.44	68.28 ± 3.07
Naya_1_	0.154	49.50 ± 7.60	22.22	74.26 ± 2.07
Naya_1_	0.308	55.79 ± 3.84	44.44	79.30 ± 0.62 °°
Siss_1_	0.003	50.73 ± 3.06	4.44	69.72 ± 1.01
Siss_1_	0.031	53.37 ± 0.02	22.22	70.94 ± 2.04
Siss_1_	0.063	53.76 ± 0.42	44.44	70.64 ± 0.02
Yate_1_	0.018	53.47 ± 0.48	4.44	64.50 ± 0.60
Yate_1_	0.184	55.15 ± 0.49	22.22	71.60 ± 0.93
Yate_1_	0.369	56.48 ± 2.27 *	44.44	80.32 ± 0.92 °°°
Crude Industrial	0.008	48.73 ± 8.37	4.44	66.60 ± 2.26
Crude Industrial	0.085	50.78 ± 7.45	22.22	70.41 ± 2.02
Crude Industrial	0.170	55.12 ± 0.86	44.44	72.79 ± 1.63
Refined Industrial	0.007	53.17 ± 1.64	4.44	68.02 ± 2.08
Refined Industrial	0.073	53.07 ± 0.17	22.22	72.42 ± 2.09
Refined Industrial	0.146	54.14 ± 0.65	44.44	77.13 ± 0.92
Kariten	0.006	46.44 ± 6.33	4.44	65.75 ± 0.02
Kariten	0.055	50.82 ± 3.40	22.22	68.80 ± 4.02
Kariten	0.110	54.61 ± 3.42	44.44	69.94 ± 0.11
TiO_2_	-	-	0.44	62.76 ± 0.79
TiO_2_	-	-	4.44	62.88 ± 6.53
TiO_2_	-	-	22.22	64.98 ± 3.28

Shows the top 3 tested polar extracts with the highest quenching effects on ^1^O_2_: *** > ** > *; Shows the top 3 tested crude shea samples with the highest quenching effects on ^1^O_2_: °°° > °° > °. Abs: Absorbance; RB: Rose Bengal; TiO_2_: Titanium dioxide; MeOH: Methanol; EtOH: Ethanol; ADPA: 9,10-Anthracenedipropanoic acid.

**Table 4:** Phenolic composition by RP-HPLC of shea butter extracts.

Sample	(1) Gallic Acid (ppm GAE)	(2) Protocatechuic Acid (ppm GAE)	(C3) Rt = 19 min (ppm GAE)	(4) Cinnamic Acid (ppm GAE)	(5) Quercetin (ppm GAE)	(C6) Rt = 54 min (ppm GAE)	(C7) Rt = 61 min (ppm GAE)	(C8) Rt = 68 min (ppm GAE)	(C9) Rt = 68 min (ppm GAE)	(C10) Rt = 69 min (ppm GAE)	(C8/C9)	(C8/C10)
Houe_5_	nd	<LOQ	0.928	9.438	nd	0.165	<LOQ	43.718	40.587	92.709	1.1	0.5
Ioba_1_	nd	<LOQ	nd	1.134	nd	0.015	<LOQ	27.046	23.646	66.189	1.1	0.4
Ioba_2_	nd	nd	nd	<LOQ	nd	1.486	<LOQ	5.226	4.001	13.184	1.3	0.4
Kadi_5_	nd	nd	nd	0.321	nd	1.549	<LOQ	40.714	35.617	82.284	1.1	0.5
Naya_1_	nd	nd	1.940	12.237	<LOQ	1.681	<LOQ	15.369	13.866	35.367	1.1	0.4
Siss_1_	nd	nd	<LOQ	0.972	nd	nd	<LOQ	22.174	19.995	46.600	1.1	0.5
Yate_1_	<LOQ	nd	0.149	2.338	<LOQ	2.014	5.282	59.585	62.168	119.037	1.0	0.5
Crude Industrial	nd	nd	0.132	46.261	nd	0.495	nd	80.292	72.885	162.707	1.1	0.5
Refined	nd	nd	nd	nd	nd	6.400	<LOQ	73.018	63.263	153.764	1.2	0.5
Kariten	3.374	nd	nd	0.961	nd	<LOD	<LOQ	12.088	10.104	29.941	1.2	0.4

LOQ: Limit of Quantification; nd: not detected; Rt = retention time in min. (1)–(2)–(4)–(5): Order numbers of phenolic compounds identified by HPLC; (C3)–(C6)–(C7)–(C8)–(C9)–(C10): Order numbers for phenolic compounds not identified by HPLC.

**Table 5:** Traditional and industrial research materials, according to their origin.

Sample	Origin
Houe_5_	Bobo-Dioulasso (Upper-Bassin) *
Ioba_1_	Dano (South West) *
Ioba_2_	Dano (South West) *
Kadi_5_	Ouagadougou (Center) *
Naya_1_	Toma (Mouhoun Loop) *
Siss_1_	Boura (Midwest) *
Yate_1_	Ouahigouya (North) *
Crude Industrial	IOI Loders Croklaan, The Netherlands
Refined Industrial	IOI Loders Croklaan, The Netherlands
Kariten-rich extract	Belgium

* Traditional shea butter from Burkina Faso.

## Data Availability

The data presented in this study are available on request from the corresponding authors.

## Supplementary Materials

The following supporting information can be downloaded at: https://www.mdpi.com/article/10.3390/molecules30061360/s1, Figure S1: Crude shea butter comparative absorbance in ethanol (a) and cyclohexane (b); Figure S2: Effects of crude shea butter on HaCaT cells cytotoxicity after 24 h of incubation (a) and 48 h of incubation (b). Cell viability was measured for 24 h and 48 h by MTS assay, and results were recorded at 3 h of three wells per sample. Ethanol was used as a control. Samples were performed at different concentrations in EtOH (µg/mL); Figure S3: (a) Chromatogram of sample Naya_1_, illustrating the separation of the SB polar extract by RP-HPLC at 280 nm, highlighted three unidentified peaks (*C8*, *C9*, and *C10*). (b) UV spectra of three non-identified peaks (*C8*, *C9*, and *C10*) from the industrial crude sample overlay to the cinnamic acid UV spectrum; Figure S4: Cellular experimental illustrative on HaCaT cells highlighted cells without RB vs. cells with RB after irradiation at λ 550 nm—*: Experimental device performing ^1^O_2_ quenching according to ADPA test for acellular model—**: Experimental device performing direct photoprotective effect for cellular model, according to MTS assay. Analysis was performed in three replicates along two independent days. Values were recorded of three wells per sample; *p* < 0.01 compared with the control; Table S1: Research material concentration related to polyphenol content.

## Abbreviations

The following abbreviations are used in this manuscript:

ABTS	3-ethylbenzothiazoline 6-sulfonic acid
ADPA	9,10-Anthracenedipropanoic acid
DMSO	Dimethyl sulfoxide
DNA	Deoxyribonucleic Acid
DPPH	2,2-Diphenyl-1-picrylhydrazyl
EtOH	Ethanol
FC	Folin–Ciocalteu
GAE	Gallic acid equivalent
HaCaT	Human keratinocyte cell line
LOQ	Limit of quantification
MeOH	Methanol
MTS	3-(4,5-Dimethylthiazol-2-yl)-5-(3-carboxymethoxyphenyl)-2-(4-sulfophenyl)-2H-tetrazolium
PGE	Pyrogallol equivalent
RB	Rose Bengal
RP-HPLC/LC	Reverse phase-High performance liquid chromatography
ROS	Reactive Oxygen Species
Rt	Retention time
SB	Shea butter
TiO_2_	Titanium dioxide
TPC	Total Polyphenol Content
UV	Ultraviolet
UVR	Ultraviolet Radiation
^1^O_2_	Singlet oxygen
