# Supplementary material for: Effects of Crude Shea Butters and Their Polar Extracts on Singlet Oxygen Quenching and Against Rose Bengal-Induced HaCaT Cell Phototoxicity
Source: Molecules. 2025 Mar 18;30(6):1360. doi: 10.3390/molecules30061360 (PMC11946392; doi:10.3390/molecules30061360)
Supplement: Supplementary file 1 [file molecules-30-01360-s001.zip › molecules-3506257-supplementary.pdf]

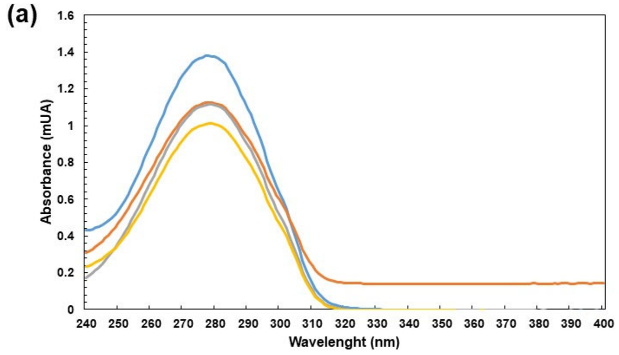

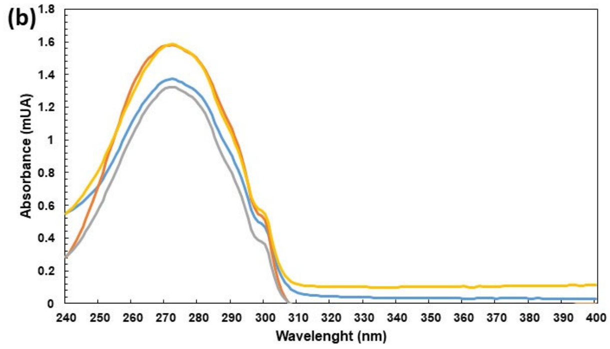

Figure S1: Crude shea butter comparative absorbance in ethanol (a) and cyclohexane (b).

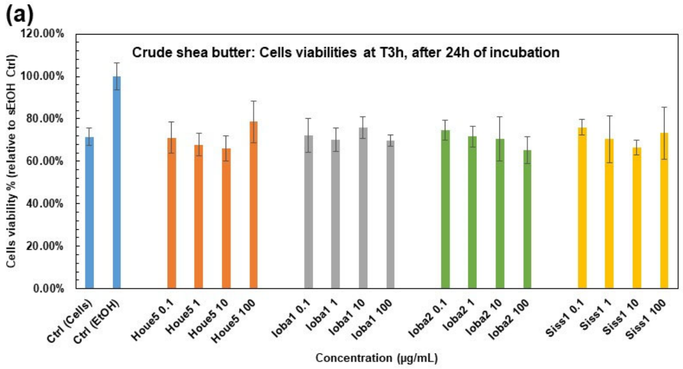

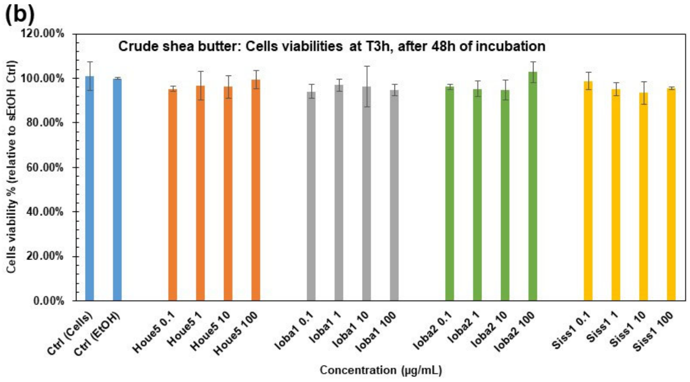

Figure S2: Effects of crude shea butter on HaCaT cells cytotoxicity after 24 h of incubation (a) and 48 h of incubation (b). Cell viability was measured for 24 h and 48 h by MTS assay, and results were recorded at 3 h of three wells per sample. Ethanol was used as a control. Samples were performed at different concentrations in EtOH (µg/mL).

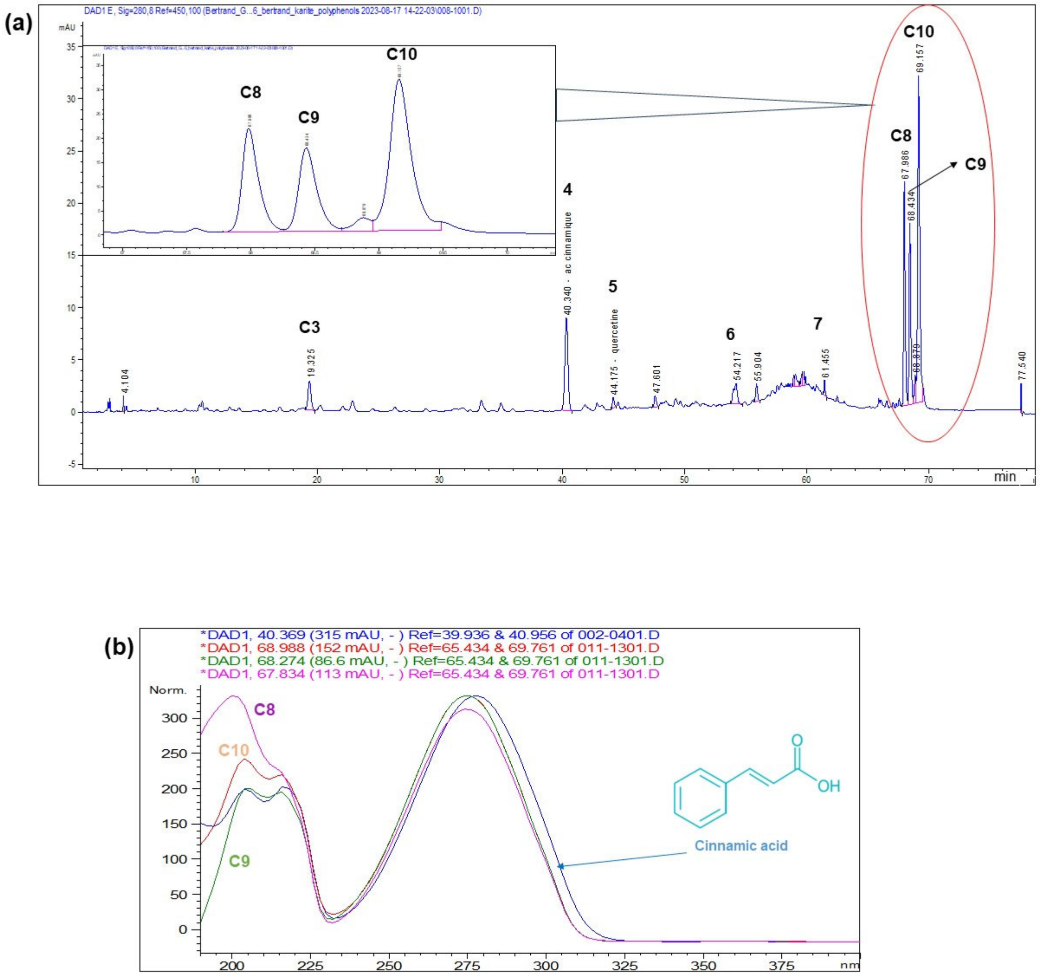

Figure S3: (a) Chromatogram of sample Naya1, illustrating the separation of the SB polar extract by RP-HPLC at 280 nm, highlighted three unidentified peaks (*C8*, *C9*, and *C10*). (b) UV spectra of three non-identified peaks (*C8*, *C9*, and *C10*) from the industrial crude sample overlay to the cinnamic acid UV spectrum.

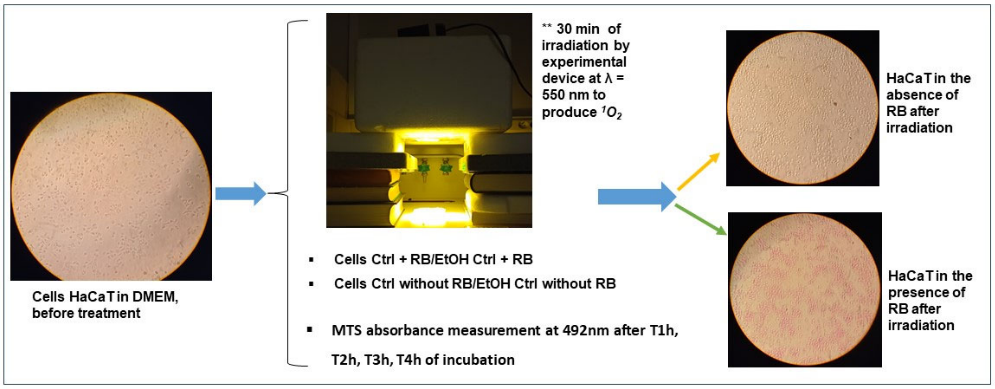

Figure S4: Cellular experimental illustrative on HaCaT cells highlighted cells without RB vs. cells with RB after irradiation at *λ* 550 nm—*: Experimental device performing $^1O_2$ quenching according to ADPA test for acellular model—**: Experimental device performing direct photoprotective effect for cellular model, according to MTS assay. Analysis was performed in three replicates along two independent days. Values were recorded of three wells per sample; $p < 0.01$ compared with the control.
