# Supplementary material for: Effects of Crude Shea Butters and Their Polar Extracts on Singlet Oxygen Quenching and Against Rose Bengal-Induced HaCaT Cell Phototoxicity
Source: Molecules. 2025 Mar 18;30(6):1360. doi: 10.3390/molecules30061360 (PMC11946392; doi:10.3390/molecules30061360)
Supplement: Supplementary file 1 [file molecules-30-01360-s001.zip › Table S1.pptx]

## Slide 1
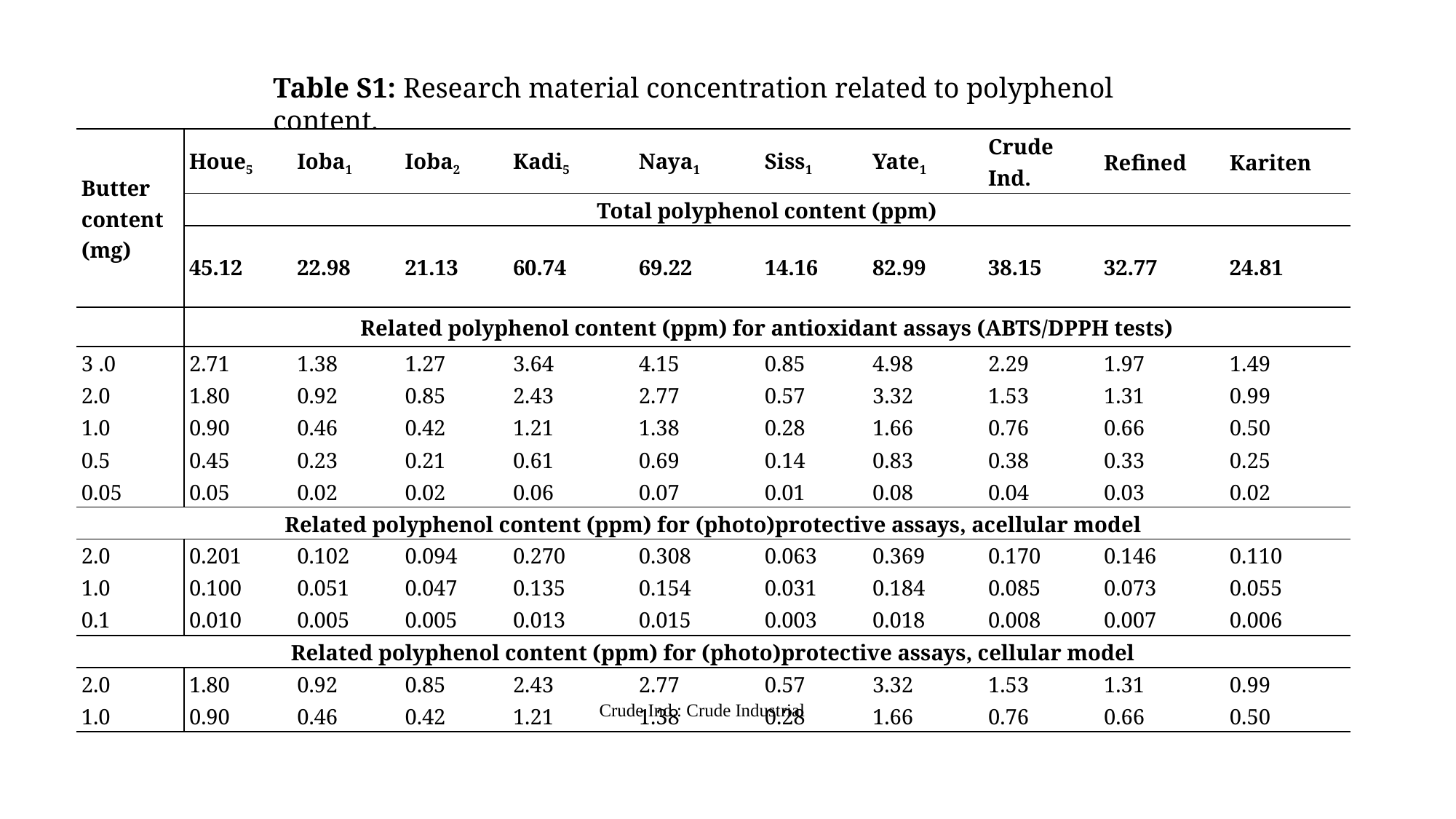

Table S1: Research material concentration related to polyphenol content.
| Butter content  (mg) | Houe5 | Ioba1 | Ioba2 | Kadi5 | Naya1 | Siss1 | Yate1 | Crude Ind. | Refined | Kariten |
| --- | --- | --- | --- | --- | --- | --- | --- | --- | --- | --- |
| | Total polyphenol content (ppm) | | | | | | | | | |
| | 45.12 | 22.98 | 21.13 | 60.74 | 69.22 | 14.16 | 82.99 | 38.15 | 32.77 | 24.81 |
| | Related polyphenol content (ppm) for antioxidant assays (ABTS/DPPH tests) | | | | | | | | | |
| 3 .0 | 2.71 | 1.38 | 1.27 | 3.64 | 4.15 | 0.85 | 4.98 | 2.29 | 1.97 | 1.49 |
| 2.0 | 1.80 | 0.92 | 0.85 | 2.43 | 2.77 | 0.57 | 3.32 | 1.53 | 1.31 | 0.99 |
| 1.0 | 0.90 | 0.46 | 0.42 | 1.21 | 1.38 | 0.28 | 1.66 | 0.76 | 0.66 | 0.50 |
| 0.5 | 0.45 | 0.23 | 0.21 | 0.61 | 0.69 | 0.14 | 0.83 | 0.38 | 0.33 | 0.25 |
| 0.05 | 0.05 | 0.02 | 0.02 | 0.06 | 0.07 | 0.01 | 0.08 | 0.04 | 0.03 | 0.02 |
| Related polyphenol content (ppm) for (photo)protective assays, acellular model | | | | | | | | | | |
| 2.0 | 0.201 | 0.102 | 0.094 | 0.270 | 0.308 | 0.063 | 0.369 | 0.170 | 0.146 | 0.110 |
| 1.0 | 0.100 | 0.051 | 0.047 | 0.135 | 0.154 | 0.031 | 0.184 | 0.085 | 0.073 | 0.055 |
| 0.1 | 0.010 | 0.005 | 0.005 | 0.013 | 0.015 | 0.003 | 0.018 | 0.008 | 0.007 | 0.006 |
| Related polyphenol content (ppm) for (photo)protective assays, cellular model | | | | | | | | | | |
| 2.0 | 1.80 | 0.92 | 0.85 | 2.43 | 2.77 | 0.57 | 3.32 | 1.53 | 1.31 | 0.99 |
| 1.0 | 0.90 | 0.46 | 0.42 | 1.21 | 1.38 | 0.28 | 1.66 | 0.76 | 0.66 | 0.50 |
Crude Ind.: Crude Industrial
